# Mixed Polymeric Micelles for Paclitaxel Delivery: Preparation and Characterization

**DOI:** 10.1021/acsomega.6c03082

**Published:** 2026-08-04

**Authors:** Gisele L. Maia, Anna Carolina S. Sousa, Marina X. Teixeira, Angelo Malachias, Maria Victoria H. Machado, Maria Betânia F. Marques, Caroline M. R. Oda, André L. B.de Barros, Elaine A. Leite

**Affiliations:** † Department of Pharmaceutical Products Faculty of Pharmacy, 28114 Federal University of Minas Gerais, Av. Antônio Carlos, 6627, Belo Horizonte 31270-901, Minas Gerais, Brazil; ‡ Departmentof Physics (ICEx), Federal University of Minas Gerais, Av. Antônio Carlos, 6627, Belo Horizonte 31270-901, Minas Gerais, Brazil; § Department of Food and Drug, Faculty of Pharmaceutical Sciences, Federal University of Alfenas, Alfenas 37130-001, Minas Gerais, Brazil; ∥ Department of Clinical and Toxicological Analyses Faculty of Pharmacy, Federal University of Minas Gerais, Av. Antônio Carlos, 6627, Belo Horizonte 31270-901, Minas Gerais, Brazil

## Abstract

To address the limitations of commercial vehicles for paclitaxel (PTX) delivery, this study investigated mixed micelles as a novel delivery system for this drug. By incorporating PCL_5000_-PEG_5000_ into DSPE-PEG_2000_ at 99.5:0.5 and 99:1 molar ratios, we aimed to enhance drug loading and biocompatibility. The resulting systems were characterized using a range of physicochemical and biological characterization. The system achieved a low critical micelle concentration (CMC) of 1.9 × 10^–5^ mol/L, ensuring stability upon dilution. Optimized formulations (99.5:0.5 and 99:1 molar ratios) exhibited a small hydrodynamic diameter (∼11 nm) and near-neutral zeta potential. Small-range X-ray scattering (SAXS) analysis confirmed a spheroidal core–shell structure, where the addition of 0.5% PCL_5000_-PEG_5000_ increased drug loading capacity to 3.1 ± 0.05% w/w. Thermal analysis confirmed the successful integration of copolymers and their thermal stability. Notably, the cytotoxicity of this formulation was comparable to that of commercial formulations, while exhibiting negligible hemolysis (<2%), indicating a substantially improved safety profile compared with Cremophor-based vehicles. These findings highlight the potential of these mixed micelles as a biocompatible, high-capacity delivery platform for intravenous cancer chemotherapy.

## Graphical abstract

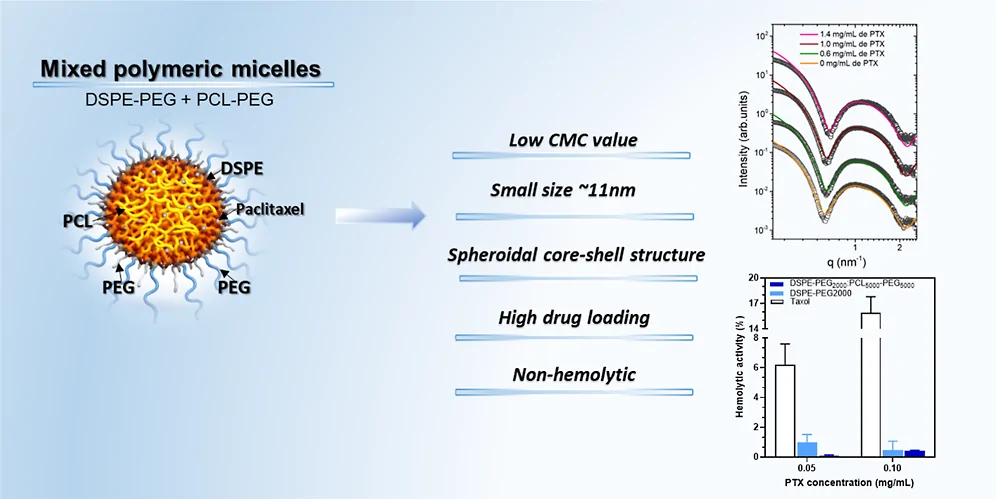

## 1 Introduction

Over the past four decades, significant progress in screening and therapeutic innovations has contributed to reducing breast cancer mortality.^1^ However, because of the persistence of high incidence and therapeutic resistance, this disease continues as an important public health concern. By 2040, the number of new breast cancer cases is estimated to increase by approximately 40%, highlighting the urgent need to develop and improve approaches for the prevention and treatment of this disease.^2^ Chemotherapy agents play a crucial role in breast cancer management, particularly in advanced or metastatic stages, and their selection is guided by the biological properties of the tumor.^3^

Paclitaxel (PTX) is a well-described anticancer drug listed on the World Health Organization Essential Medicines List for the treatment of early stage and metastatic breast cancer. Given its established mechanism of action, numerous investigations have shown that the antitumor activity of PTX is achieved through interconnected mechanisms, including microtubule stabilization, which disrupts mitotic progression, and activation of signaling cascades that culminate in apoptosis.^4,5^ Besides, PTX has been reported to influence the expression of microRNAs linked to tumor progression, to contribute to immune modulation by altering cytokine and chemokine profiles, and to modulate immune cell dynamics.^6^ However, PTX has an extremely low aqueous solubility, requiring solvent-based formulations. Conventional formulations such as Taxol employ Cremophor EL and ethanol as solubilizing agents, which are associated with severe hypersensitivity reactions, neurotoxicity, nephrotoxicity, and altered pharmacokinetics, frequently requiring premedication prior to administration.^7^ ^,^

Recent innovations such as albumin-bound paclitaxel (Abraxane), polymeric micelles (Genexol-PM), and liposomal formulations (Lipusu) approved for clinical use in different countries exemplify the advancement of nanotechnology-based approaches designed to enhance the anticancer efficacy and reduce side effects. Despite avoiding Cremophor-related toxicity, these formulations still resulted in significant side effects. A recent systematic review and network meta-analysis demonstrated that Abraxane exhibited higher incidences of neutropenia and leukopenia than solvent-based paclitaxel formulations, although the incidence of peripheral neuropathy was lower than that observed with Taxol.^8^ These findings highlight that despite the advances achieved with formulations such as Abraxane, challenges associated with safety treatment-related toxicities remain. Therefore, alternative delivery systems capable of accommodating higher PTX payloads while maintaining long-term physicochemical stability and favorable biological performance are still needed.

In this context, our research group developed distearoylphosphatidylethanolamine–polyethylene glycol (DSPE-PEG_2000_) polymeric micelles containing PTX, which exhibited promising physicochemical and biological properties for tumor targeting and enabled theranostic use in breast cancer via 99 mTc-radiolabeling.^9^ Additionally, this formulation demonstrated better performance than Taxol, as evidenced by its high efficacy and the absence of peripheral neuropathy. However, limitations in long-term physicochemical stability were observed, highlighting the need for further optimization.^10,11^

Mixed polymeric micelles have emerged as a promising strategy, combining different amphiphilic polymers to increase hydrophobic interactions within the micellar core, thereby enhancing the loading capacity and stability of hydrophobic drugs.^12,13^

Based on this rationale, the mixed polymeric micelles reported herein, composed of DSPE-PEG_2000_ and a poly­(ε-caprolactone) copolymer coupled to polyethylene glycol (PCL_5000_-PEG_5000_), address limitations associated with commercial PTX formulations and our previously developed DSPE-PEG_2000_ micelles. The incorporation of PCL_5000_-PEG_5000_ into the micellar structure was expected to enhance PTX loading capacity and improve micellar stability while preserving the biocompatibility and stealth properties conferred by PEGylation. This copolymer was selected because PCL-PEG micelles have been extensively investigated for the delivery of anticancer drugs. Recent reviews have highlighted the versatility of PCL-based nanocarriers and their continued development as promising platforms for cancer nanomedicine and controlled drug-delivery applications.^14–16^ Thus, the formulations were characterized for their physicochemical properties (particle size, zeta potential, and encapsulation efficiency) and subsequently assessed for stability. Furthermore, thermal analyses and small-angle X-ray scattering (SAXS) were performed to infer intermolecular interactions between PTX and the polymers, providing insights into the structural mechanisms governing micelle formation, stability, and drug retention.

## 2 Materials

### 2.1 Chemicals

DSPE-PEG_2000_ was purchased from Lipoid GmbH (Ludwigshafen, Germany). PCL_5000_-PEG_5000_ was obtained from Sigma-Aldrich (Missouri, EUA). PTX was provided by Quiral Química do Brasil S/A (Juiz de Fora, Brazil) with a purity greater than 97%. Sodium chloride P.A. was purchased from Merck (Darmstadt, Germany). Sodium salicylate P.A. was obtained from Dinâmica (Indaiatuba, Brazil). Ultrapure water was produced using a Milli-Q purification system (Millipore, USA). Cremophor EL was purchased from Sigma-Aldrich Co. (Missouri, USA). All other reagents used were of analytical grade or higher.

## 3 Experimental Section

### 3.1 Critical Micellar Concentration Determination

The critical micelle concentration (CMC) of the DSPE-PEG_2000_ and PCL_5000_-PEG_5000_ mixture at a 90:10 molar ratio was determined at 25 °C using a fluorescent probe method previously reported.^11^ Aliquots of DSPE-PEG_2000_ (10 mmol L^–1^) and PCL_5000_-PEG_5000_ (0.1 mmol L^–1^) in chloroform were combined in a round-bottom flask, and the solvent was removed by reduced pressure to form a thin polymer film. This film was hydrated with phosphate-buffered saline (PBS, pH 7.4). Aliquots of the dispersion were mixed with 1 mg of pyrene, adjusting the volume with PBS to achieve polymer concentrations between 10^–4^ and 10^–6^ mol L^–1^. After overnight incubation at room temperature with stirring, free pyrene was removed by filtration through 0.22 μm polycarbonate membranes. Fluorescence measurements using a Cary Eclipse spectrometer (Varian, Inc., USA) at excitation and emission wavelengths of 339 and 390 nm, respectively, were determined. CMC value was determined graphically as the intersection of the two straight lines obtained by plotting fluorescence intensity dependency on DSPE-PEG_2000_: PCL_5000_-PEG_5000_ relative concentration.

### 3.2 Mixed Polymeric Micelles’ Preparation

Mixed polymeric micelles were prepared by solvent evaporation, as described by Oda et al. (2017), with modifications.^11^ Briefly, an aliquot of PCL_5000_-PEG_5000_ in chloroform was transferred to a round-bottom flask, and the solvent was removed under vacuum (55 °C, 150 rpm, 131 mbar) until a thin, uniform film was formed. Then, the DSPE-PEG_2000_ solution, with or without paclitaxel (PTX), was added to a round-bottom flask to obtain the desired formulation (99:10, 99:1, or 99.5:0.5 molar ratio). After vortex mixing to solubilize the film, the solvent was again evaporated under the same conditions. The resulting film was hydrated with preheated 0.9% (w/v) NaCl solution at 55 °C, incubated in the water bath (55 °C) for 5 min, and vortexed to ensure complete dispersion. The formulations were cooled to room temperature and then subjected to a thorough purification process, involving centrifugation (6500*g*, 15 min) and filtration through a 0.22 μm membrane to remove nonencapsulated drug.

### 3.3 Nanoparticle Characterization

All formulations were prepared in three independent batches. Physicochemical characterization was performed for each batch, and the results are reported as the mean ± standard deviation (SD) of independent preparations.

#### 3.3.1 Average Diameter, Polydispersity Index, and Zeta Potential Measurements

For average diameter and PDI determination, dynamic light scattering (DLS) at 25 °C and a 90° (unimodal analysis) was used. Zeta potential was evaluated by DLS with electrophoretic mobility at 90° and 25 °C after 100-fold dilution in an aqueous solution of 1 mmol/L NaCl (w/v), previously filtered through a 0.45 μm filter. Measurements were performed on at least three independent batches using a Zetasizer Nano ZS90 (Malvern Instruments Ltd., Malvern, England). Data are expressed as mean ± standard deviation (SD).

#### 3.3.2 Encapsulation Efficiency

Encapsulated PTX was quantified by high-performance liquid chromatography (HPLC) following the same protocol and conditions previously described.^17^ Encapsulation efficiency (EE) was calculated according to eq 1:

1 %EE=(Druginpurifiednanoparticles/Druginnon‐purifiednanoparticles)×100

#### 3.3.3 Small-Angle X-ray Scattering (SAXS) Characterization

Structural information, such as the shape and size of micelles, was obtained via SAXS analysis using SAXS-1 beamline of the Brazilian Synchrotron Light Laboratory (LNLS, Campinas, Brazil). The analysis was performed using a collimated beam with a fixed wavelength of 1.57 Å (8 keV) with the Pilatus 300 K detector positioned 1.5 m from the sample, providing a *q*-range from 0.05 to 3 nm^–1^. Experiments were conducted at room temperature and injected into a temperature-controlled cell consisting of two mica windows separated by 1 mm, under vacuum.

Two-dimensional images obtained by the detector were normalized to the incident beam intensity and sample absorption. Background scattering from the sample cell (wirg mica windows) and solvent (0.9% w/v NaCl solution) was subtracted, and the resulting data were converted into scattering intensity profiles *I*(*q*) as a function of scattering vector *q* using Fit2D software.

Finally, the scattering patterns were fitted using a mathematical model based on the scattering intensity of spherical polymeric micelles formed by diblock copolymers, as proposed by Pedersen and Gerstenberg (1996).^18^

#### 3.3.4 Evaluation of Thermal Properties

The thermal characteristics of pharmaceutical ingredients and mixed polymeric micelle formulations were studied using differential scanning calorimetry (DSC) and thermogravimetric analysis (TGA). DSC analyses were conducted in a Shimadzu DSC-60 plus calorimeter, previously calibrated with Indium (*T*m_onset_ 156.63 °C, Δ*H*m = 28.45 J g^–1^), under a dynamic nitrogen atmosphere (50 mL min^–1^). Approximately, 1.5 mg of the sample was accurately weighed and sealed in an aluminum crucible, and the measurements were carried out over the 30–400 °C temperature range at a heating rate of 10 °C min^–1^.

For TGA and differential thermal analyses (TGA/DTA), the measurements were performed using a Shimadzu DTG-60H thermobalance under a nitrogen flow of 50 mL min^–1^, with a heating rate of 10 °C min^–1^ over the 30–600 °C range. Each measurement employed approximately 2.5 mg of the sample placed in an open alumina crucible. The first derivative of the TG curve (DTG) was obtained using the instrument’s TA60 software version 2.30 SP1 to confirm the occurrence of thermal events, quantify the mass losses (Δ*m*), and determine the corresponding decomposition temperatures and other thermal transitions.^19–21^

#### 3.3.5 Stability Study

The storage stability of the formulations in aqueous medium was evaluated by monitoring physicochemical parameters, including mean diameter, zeta potential, and drug retention. The samples were stored at 4 °C and analyzed after 1, 7, 15, 30, and 60 days of storage. At each time point, the formulations were filtered through a 0.22 μm membrane and compared with freshly prepared samples (day 0). All measurements were performed in triplicate, and results were expressed as mean ± standard deviation.

Colloidal stability was also evaluated after exposing formulations to fetal bovine serum (FBS) and RPMI 1640 cell culture medium. The dispersions were maintained at 37 ± 2 °C with agitation for 24 h. At predetermined time intervals (1, 2, 4, 8, 24 h), changes in the diameter were evaluated by DLS.

#### 3.3.6 In Vitro Release Study

The in vitro release profiles of DSPE-PEG_2000_ micelles (0.6 mg/mL PTX) and mixed DSPE-PEG_2000_/PCL_5000‑_PEG_5000_ micelles (1 mg/mL PTX, 99.5:0.5 molar ratio) were evaluated in an aqueous medium containing 1 mol/L sodium salicylate, as described by Cho et al. (2004).^22^ The solubility of PTX in this medium was determined by incubating an excess amount of the drug in 2 mL of 1 mol/L sodium salicylate solution under stirring (120 rpm) at 37 °C for 24 h. The samples were filtered through 0.22 μm membranes, and the dissolved PTX concentration was quantified by HPLC as previously described.

For the release study, 0.5 mL of each freshly prepared formulation was placed into dialysis membranes (MWCO = 14,000 Da, Sigma-Aldrich, USA), sealed, and immersed in 50 mL of 1 mol/L sodium salicylate solution at 37 °C. The system was maintained under stirring at 120 rpm using an IKA KS 4000i Control shaker (Werke, Germany). At predetermined intervals (30 min, 1 h, 2 h, 4 h, 8 h, 24 h, and 48 h), 0.5 mL aliquots were withdrawn and replaced with fresh medium at the same temperature. The concentration of released PTX was determined by HPLC, and all analyses were performed in triplicate. Results are expressed here as mean ± standard deviation.

#### 3.3.7 Cytotoxicity Study

The cytotoxicity was measured using the sulforhodamine B (SRB) assay.^23^ The murine breast cancer cell line 4T1 (ATCC CRL-2539) was used to evaluate the antitumor activity of formulations at passage XX and maintained in a cell incubator at 37 °C with 5% CO_2_. The 4T1 cells were cultured in T-75 flasks using RPMI 1640 supplemented with 10% (v/v) heat-inactivated FBS, penicillin (100 IU/mL), and streptomycin (100 μg/mL). After reaching confluency of 80–90%, the cells were harvested by trypsinization, and the viable cell count was performed using a Neubauer hemocytometer with trypan blue (1:1) dye exclusion. Then, 4T1 cells were seeded (5 × 10^4^/well in 96-well plates), and after one day at 37 °C in a 5% CO_2_, humidified atmosphere, cells were exposed to DSPE-PEG_2000_:PTX, DSPE-PEG_2000_:PCL_5000_-PEG_5000_:PTX, or Taxol diluted with complete RPMI 1640 medium to reach concentrations ranging from 2.5 μM to 0.6 nM. Subsequently, the cells were incubated for 48 h and then the SRB assay protocol previously described was carried out.^23^ Cell density was determined by measuring absorbance at 510 nm with a SpectraMax Plus 384 microplate spectrophotometer (Molecular Devices, Sunnyvale, USA), and cell viability was determined as the percentage of absorbance relative to untreated cells used as a control. The half-maximal inhibitory concentration (IC_50_) was calculated using GraphPad Prism software by nonlinear regression of concentration–response curves with a variable-slope model. The percentage inhibition was plotted against the logarithm of compound concentration, and the data were fitted to a sigmoidal dose–response equation.

### 3.4 Hemolysis Assay

Hemolytic activity was assessed according to published procedures.^10,24^ Blood was collected from female Swiss mice, 8 weeks old, 20.0 ± 2.0 g (Ethics Committee on Animal Use—CEUA from UFMG, protocol number 148/2017) in tubes containing 10% (w/v) EDTA. Red blood cells (RBCs) were isolated by centrifugation at 3000 rpm for 10 min at room temperature and washed repeatedly with 0.9% (w/v) NaCl until the supernatant was clear. The final RBC suspension was prepared at a concentration of 4% (w/v) in 0.9% NaCl. Taxol (Cremophor:ethanol:PTX), DSPE-PEG_2000_:PTX, and DSPE-PEG_2000_:PCL_5000_-PEG_5000_:PTX formulations were evaluated at PTX concentrations of 0.05 and 0.10 mg/mL, along with their respective controls. Each formulation was incubated with an equal volume of RBC suspension (*n* = 5) for 1 h at 37 °C under agitation (500 bpm). After incubation, samples were centrifuged at 2000 rpm for 5 min, and the absorbance of the supernatants was measured at 540 nm using a UV–visible spectrophotometer. Deionized water and 0.9% NaCl were used as positive and negative controls, respectively. Hemolysis percentages were calculated using the following equation:

%hemolysis=sampleabsorbance−negativecontrolabsorbance/positivecontrol−negativecontrolabsorbance×100

### 3.5 Statistical Analysis

All formulations were prepared at least in triplicate as independent batches, and the data are expressed as mean ± standard deviation (SD). Normality and homoscedasticity were assessed using the Shapiro–Wilk and Brown–Forysthe tests, respectively. Variables that did not follow a normal distribution were transformed, when necessary, according to the equations *y* = (variable^2^) or *y* = (1/variable). Differences between groups were evaluated using Student’s *t*-test or one-way ANOVA followed by Tukey’s post hoc test. A 95% confidence interval was adopted, and differences were considered statistically significant when the *p*-value was less than or equal to 0.05 (*p* ≤ 0.05). All analyses were performed using GraphPad Prism version 8.0.

## 4 Results and Discussion

### 4.1 CMC Determination

A key parameter for obtaining micellar formulations is the determination of the critical micellar concentration (CMC), which corresponds to the concentration above which the association of amphiphilic units in solution begins. The fluorescence intensity was measured for ten different concentrations of DSPE-PEG_2000_: PCL_5000_-PEG_5000_ at 90:10 molar ratio. A plot of fluorescence intensity versus concentration was then constructed (Figure 1), and the CMC was determined from this curve as the concentration corresponding to the end of the first plateau of the fluorescence intensity, yielding a value of 1.9 × 10^–5^ mol L^–1^. Previous study reported a CMC of 1.8 × 10^–5^ mol/L for DSPE-PEG_2000_ micelles using the same fluorescence method with pyrene as a probe.^11^ Thus, the association of DSPE-PEG_2000_ and PCL_5000_-PEG_5000_ at a 90:10 molar ratio yields a CMC similar to that of DSPE-PEG_2000_ alone. Micellar systems composed of DSPE-PEG_2000_ exhibit low CMC values due to the strong hydrophobic driving force for monomer self-assembly, which arises from the presence of two saturated acyclic 18-carbon chains.^25,26^ In general, the low CMC values of copolymers (typically 0.1–1 μmol/L), compared with those of conventional surfactants (typically 0.1–1 mmol/L), positively impact the formation of polymeric micelles, as the thermodynamically driving aggregation of monomers is favored.^27,28^

**Figure 1:**
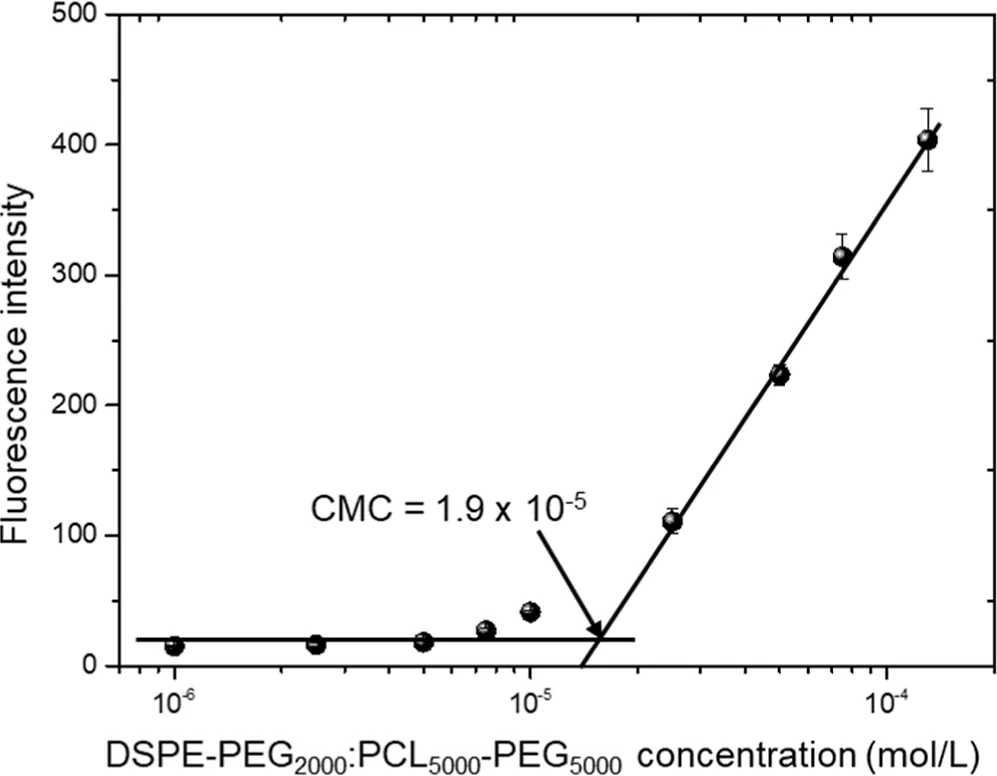
Pyrene fluorescence intensity at 390 nm as a function of DSPE-PEG_2000_ and PCL_5000_-PEG_5000_ (90:10 molar ratio) concentration highlights the CMC, the inflection point at which the fluorescence intensity begins to rise. Solid lines are shown as a visual guide, and symbol sizes are larger than the error bars for some fluorescence intensity values.

### 4.2 Formulation Development and Physicochemical Characterization

On the basis of CMC data and to ensure robustness upon dilution, a total concentration of 10 mmol/L of DSPE-PEG_2000_:PCL_5000_-PEG_5000_ was selected in this study, corresponding to approximately 500× the CMC value. This definition is important because after intravenous administration, micellar systems are subjected to extensive dilution. Such dilution may compromise micelle stability, leading to premature disassembly and leakage of the encapsulated drug before reaching the target site.^29,30^

A schematic representation of the design concept for the micelle organization is presented in Figure 2. Mixed micelles, both blank and paclitaxel (PTX)-loaded, were prepared by using DSPE-PEG_2000_ and PCL_5000_-PEG_5000_ at a molar ratio of 90:10. Both formulations exhibited a mean hydrodynamic diameter of approximately 13 nm and ζ-potential near the neutral range.

**Figure 2:**
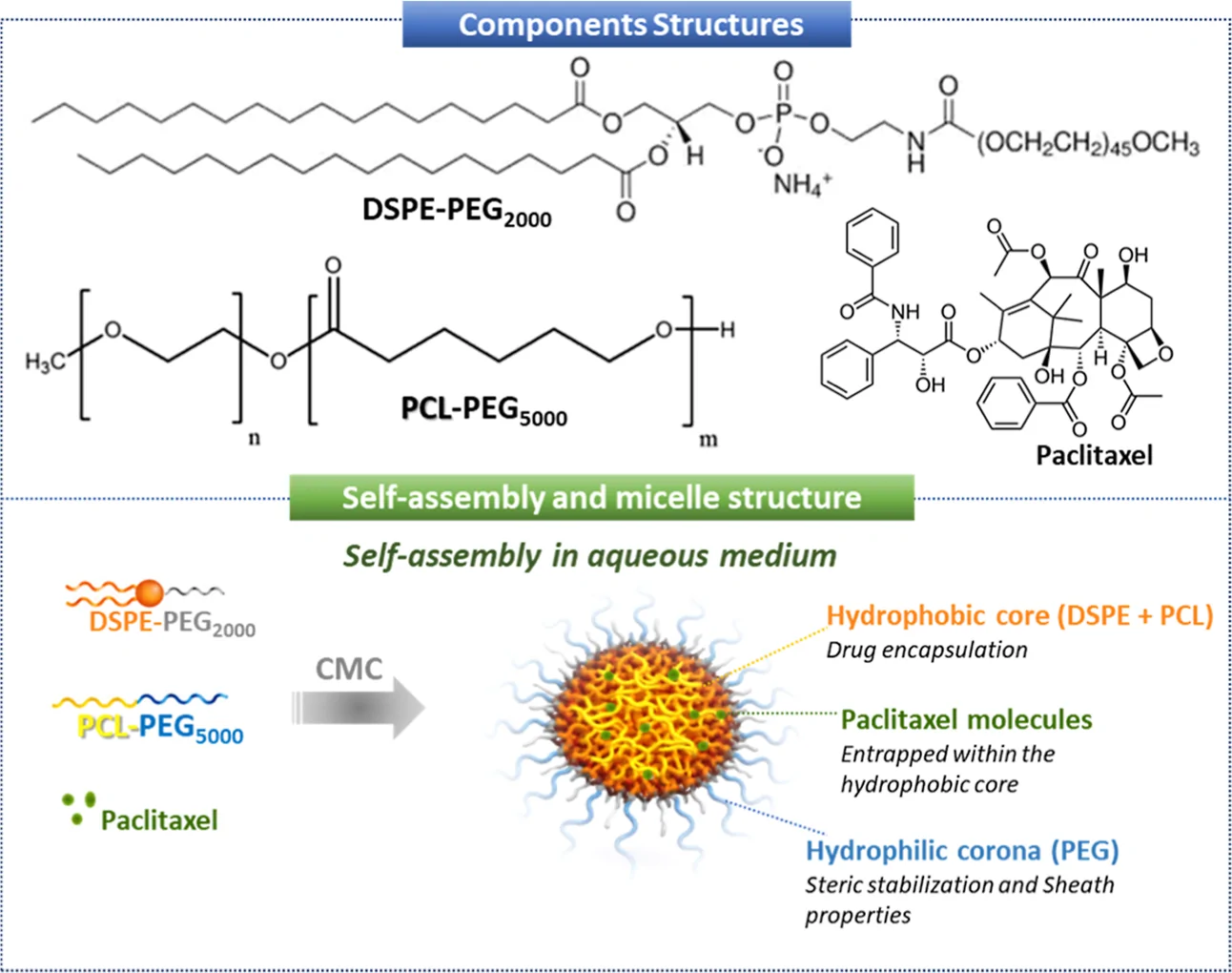
Schematic illustration of the design concept of paclitaxel-loaded mixed polymeric micelles. DSPE-PEG_2000_ and PCL_5000_-PEG_5000_ self-assemble in aqueous media to form mixed micelles consisting of a hydrophobic core and a PEG hydrophilic corona. PTX is encapsulated within the hydrophobic core.

The PTX-loaded micelles achieved an encapsulation efficiency of 72% with a drug concentration of 0.91 mg/mL. However, during preparation, either for the blank or with PTX, a high level of precipitation was observed. This precipitation was attributed to PCL_5000_-PEG_5000_, as no precipitation occurred with DSPE-PEG_2000_ micelles. On the basis of these results, the DSPE-PEG_2000_:PCL_5000_-PEG_5000_ molar ratio was changed to 99:1 and 99.5:0.5. Eight formulations were produced by varying the molar ratio of PCL_5000_:PEG_5000_ and the PTX concentration. Results from the physicochemical characterization are presented in Table 1.

**Table 1:** Diameter, Zeta Potential, PTX Concentration, and Encapsulation Efficiency of Mixed Micelles Composed of DSPE-PEG_2000_:PCL_5000_:PEG_5000_ at Molar Ratios of 99.5:0.5 and 99:1^a^

theoretical PTX concentration (mg/mL)	diameter (nm)	zeta potential (mV)	PTX concentration (mg/mL)	encapsulation efficiency (%)
99.5:0.5				
0	10.8 ± 1.4	–4.3 ± 1.5	-	-
0.6	11.9 ± 0.7	–5.3 ± 0.6^d,*^	0.4 ± 0.1^a,c,d^	72.1 ± 7.8^a^
1.0	11.0 ± 0.3	–3.2 ± 1.3	0.9 ± 0.1^a,b^	91.1 ± 3.6^a,d^
1.4	11.8 ± 2.5	–2.5 ± 0.2^b^	0.9 ± 0.1^a,b^	63.9 ± 13.5^a,c^
99:1				
0	9.2 ± 1.9	–5.0 ± 1.4	-	-
0.6	11.0 ± 1.3	–7.2 ± 0.4^c,d,*^	0.4 ± 0.1^a,c,d^	79.7 ± 15.3^a^
1.0	10.3 ± 0.8	–3.1 ± 0.2^b^	0.8 ± 0.1^a,b^	87.3 ± 5.1^a,d^
1.4	10.1 ± 2.0	–2.8 ± 0.6^a,b^	0.9 ± 0.2^a,b^	64.4 ± 11.1^a,c^

a Data are expressed as mean ± standard deviation (*n* = 3). The letters a, b, c, and d indicate, for the same PCL_5000_:PEG_5000_ molar ratio, a significant difference in the parameter when compared with formulations containing 0 mg/mL, 0.6 mg/mL, 1.0 mg/mL, and 1.4 mg/mL of PTX, respectively, as determined by one-way ANOVA followed by Tukey’s post hoc test. The asterisk indicates a significant difference between the formulations with molar ratios 99.5:0.5 and 99:1, at a fixed theoretical PTX concentration, as determined by Student’s *t*-test. The significance level was set at *p* ≤ 0.05.

The formulations exhibited an average diameter of 10.7 ± 1.4 nm, similar to those previously reported for DSPE-PEG_2000_ micelles containing 0.6 mg/mL of PTX (10.7 ± 0.8 nm).^10,31^ Other studies in the literature have reported diameter ranges between 7 and 35 nm for DSPE-PEG_2000_ micelles.^25,32–35^ Variations in the PCL_5000_:PEG_5000_ molar ratio, as well as changes in the added drug concentration, did not significantly affect the diameter of the mixed micelles. In nanoformulations intended for antitumor applications, particle diameter plays a crucial role in determining factors such as biodistribution, tumor penetration, cellular internalization, and plasma and tissue clearance, thereby exerting a significant impact on therapeutic efficacy.^36–38^ Previous studies indicated that nanoparticles with diameters close to or smaller than 50 nm exhibit improved in vivo performance, including more efficient cellular internalization, enhanced tissue penetration, and greater tumor inhibition.^38,39^ The results obtained in the present study are, therefore, favorable for a drug delivery system intended for antitumor applications. Oda et al. also reported prolonged circulation and preferential accumulation in the tumor region in an in vivo murine breast cancer model, using a nanostructured system with an analogous diameter.^10,11^

Concerning the zeta potential, an average value of −4.2 ± 1.7 mV was obtained. Low zeta potential values have been described for PEGylated particle systems and may be related to the increased thickness of the aqueous layer surrounding the particles. The presence of PEG chains on the surface shifts the hydrodynamic shear plane away from the charge plane, thereby reducing electrophoretic mobility and resulting in a zeta potential closer to neutrality.^40–42^ Furthermore, a study by Shi et al. suggested a direct relationship between the increase in PEG chain length on the surface and a more pronounced decrease in zeta potential.^42^ Nanosystems with positive surface charge, or those produced with cationic polymers, may bind more readily to the negatively charged surface of vascular endothelial cells. This interaction may lead to a rapid decrease in the nanosystem’s plasma concentration, a lower AUC, and consequently reduced drug accumulation in the tumor region via the enhanced permeability and retention effect. In contrast, negatively charged nanosystems are expected to exhibit longer plasma half-life due to the lower likelihood of uptake by the mononuclear system.^43,44^

As shown in Table 1, when the drug concentration was kept constant, no significant differences were identified among the formulations in the PCL_5000_-PEG_5000_ molar ratio from 0.5% to 1.0%. However, significant differences were observed with increasing added PTX concentration. The highest encapsulation efficiencies were found for the formulations with a PTX concentration of 1.0 mg/mL. The incorporation of PCL_5000_-PEG_5000_ increased the PTX loading capacity of the mixed micellar systems (3.1 ± 0.05% and 2.7 ± 0.08% w/w for the system composed of DSPE-PEG_2000_:PCL_5000_-PEG_5000_ at a molar ratio of 99.5:0.5 and 99:1, respectively), compared with the system composed solely of DSPE-PEG_2000_ (2.1 ± 0.10% w/w).^10^ Statistical analysis demonstrated that both PCL_5000_-PEG_5000_-containing formulations exhibited significantly higher PTX loading capacities than the DSPE-PEG_2000_ micelles (p equal to 0.0246 and 0.0021, respectively), indicating that the incorporation of PCL_5000_-PEG_5000_ enhanced PTX encapsulation.^10^ Gao et al. and Dabholkhar et al. also observed an increase in this parameter upon adding a second polymeric component, obtaining 4.0% w/w for the DSPE-PEG_2000_:EPC system (molar ratio 1:4) and 3.5% w/w for the DSPE-PEG_2000_:TPGS system (molar ratio 1:1), respectively.^45,46^

The improvement in the loading capacity of mixed micellar systems as carriers of poorly soluble drugs is associated with the incorporation of hydrophobic segments that are compatible and capable of promoting more favorable interactions between drug molecules and the micellar core.^47^

### 4.3 SAXS Analysis

The SAXS technique was employed to investigate the structural organization of the mixed polymeric micelles. The influence of PCL_5000_-PEG_5000_ and the effect of PTX concentration in the formulations were evaluated. In this study, the hydrophobic core, composed of the DSPE and PCL segments, can accommodate PTX molecules, whereas the hydrophilic shell, composed of PEG segments with varying chain lengths depending on the raw materials used, stabilizes the micelle in aqueous media. The small-angle X-ray scattering patterns obtained for the samples, along with the fits using a scattering model for spheroidal shaped micelles with core–shell structure (solid lines), are shown in Figure 3. This core–shell organization model was previously reported by Akiba et al.^48^ and Oda et al.^31^ The well-defined first local minimum position observed along the *q*-axis (between 0.61 and 0.68 nm^–1^) for all scattering curves indicates a narrow size distribution and enables a rapid estimation of the micelle diameter using the relationship *D* = 2π/*q*.

**Figure 3:**
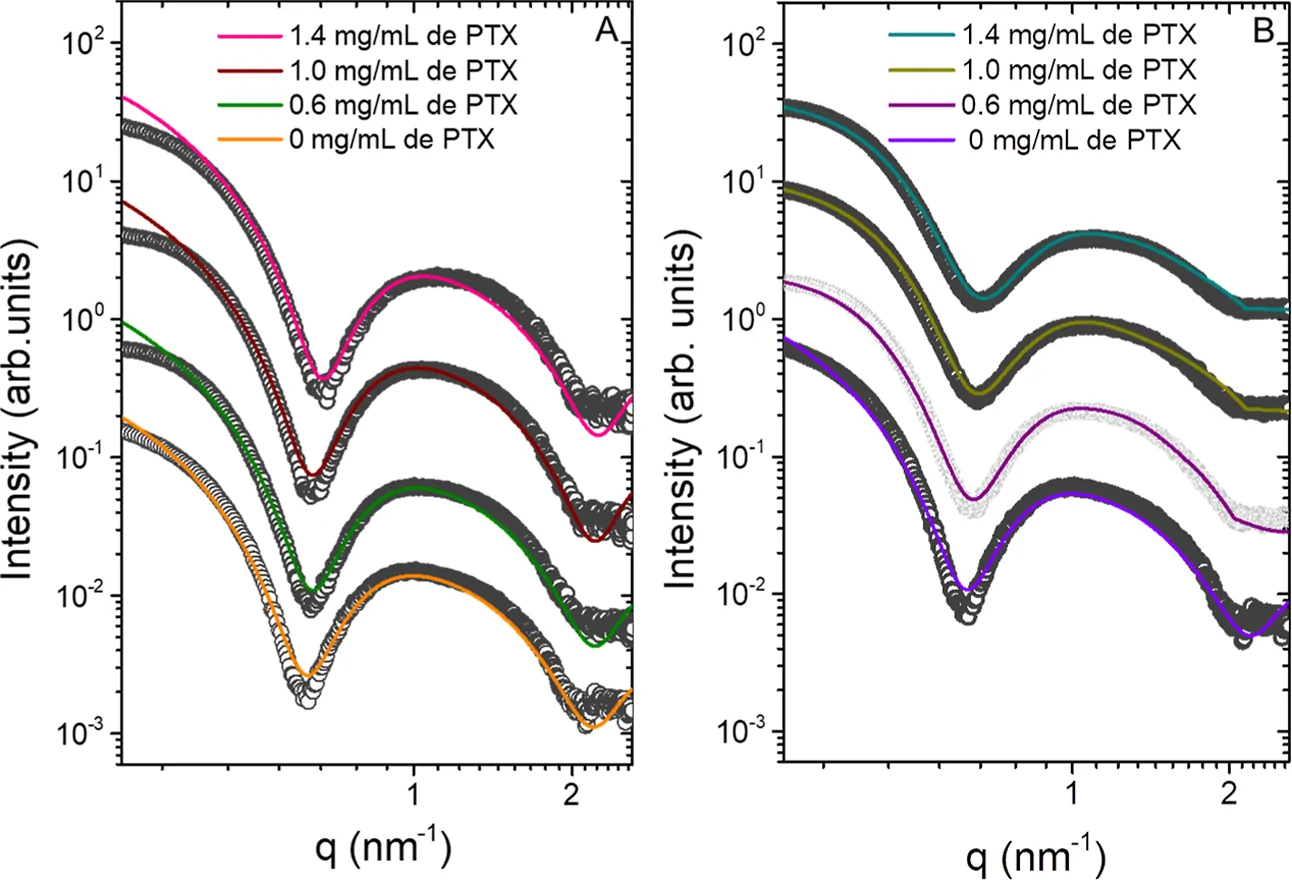
Structural analysis of mixed micelles containing PTX. SAXS patterns of mixed micelles composed of DSPE-PEG_2000_:PCL_5000_-PEG_5000_ at molar ratios of 99.5:0.5 (A) and 99:1 (B), prepared with different PTX concentrations, along with their respective curve fittings (solid lines).

Fits carried out to the data sets of Figure 2A,B show a mild variation in micelle diameter and center-to-center distance as a function of the increasing PTX concentration. It can be seen that for all formulations evaluated, the diameter remained close to 10 nm. This value is consistent with the previously obtained DLS value (approximately 11 nm, see Table 1). Such result indicates that the presence of PCL_5000_-PEG_5000_ at 0.5% and 1.0% molar ratios, and distinct PTX concentrations, did not significantly affect the size of the mixed micelles.

Regarding the center-to-center distance parameter (d) extracted from the fits [fully described at ref 31], no significant change was observed in mixed micelles prepared with 0.5% PCL_5000_-PEG_5000_ at increasing PTX concentrations (Figure 4A), with d-values ranging from 17.6 to 19.1 nm. In contrast, the d-values consistently decrease from 19.1 to 12.9 nm with increasing PTX concentrations in the mixed micelle formulations containing 1.0% PCL_5000_-PEG_5000_ (Figure 4B), suggesting micelle approximation. Additionally, alterations in the surface packing parameter (α) and the PEG chain surface density (σ) were evaluated (Figure 4C,D). The parameter α describes the conformational regime of the shell chains, and values of α < 1 indicate a more compact molecular arrangement compared to isolated (noninteracting) molecular length. The parameter σ denotes the fraction of the shell surface effectively covered by PEG. When σ ≥ 1, the interface becomes fully covered and the chains remain isolated from each other. For σ < 1, the core interface is only partially covered. Further increases in σ lead to overcrowding, resulting in more extended chain conformations.^49^ For all formulations, regardless of the PCL_5000_-PEG_5000_ molar ratio, the packing parameter values were below 1, while the surface density parameter values were above 1, indicating that the PEG chains were in a compact regime and that the shell region was fully covered.

**Figure 4:**
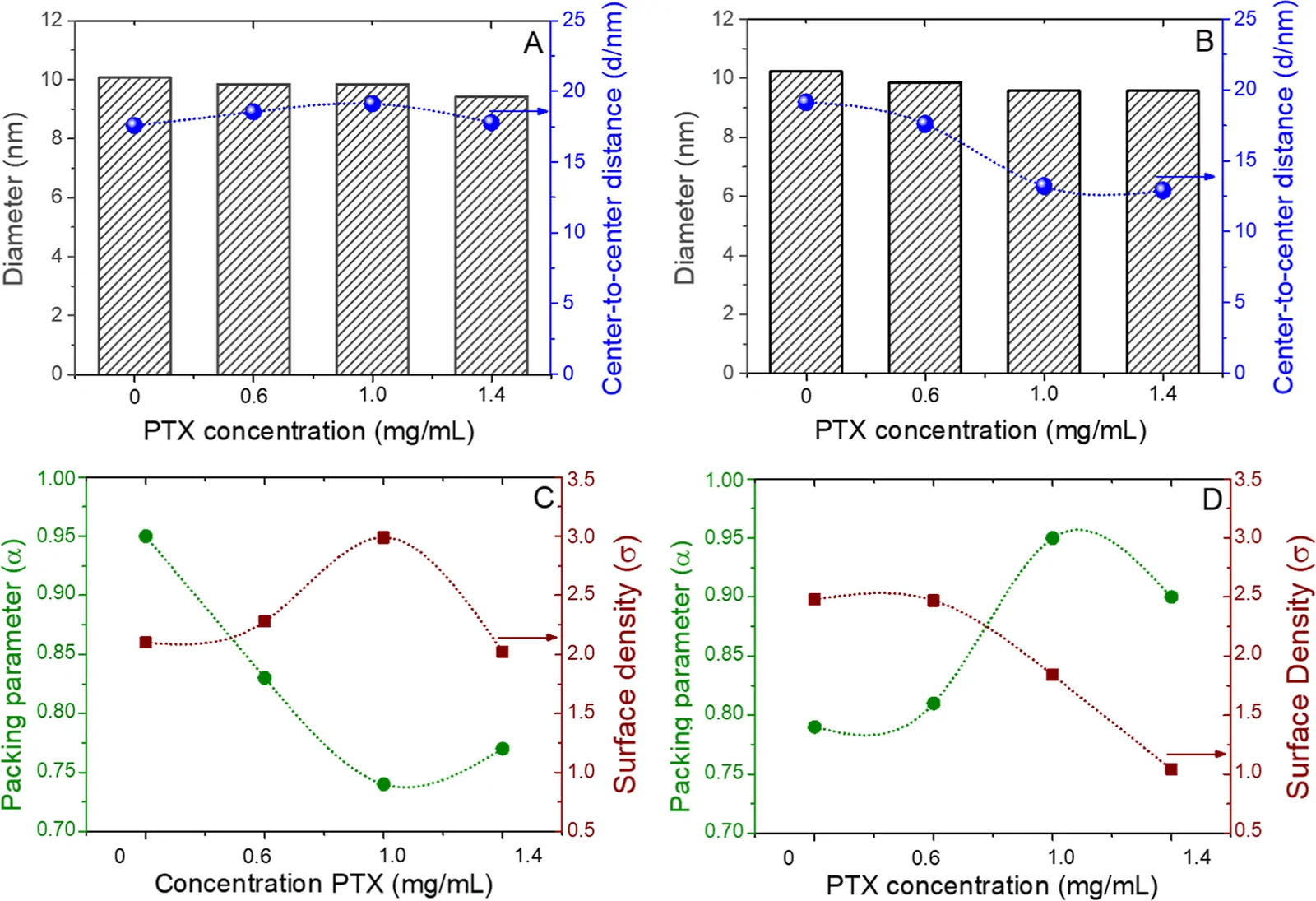
Structural parameters of mixed DSPE-PEG_2000_:PCL_5000_-PEG_5000_ micelles at increasing PTX concentrations. Panels A–B show changes in micellar diameter and center-to-center distance for molar ratios of 99.5:0.5 (A) and 99:1 (B). Panels C–D depict corresponding variations in shell packing (α) and surface density (σ) for molar ratios of 99.5:0.5 (C) and 99:1 (D).

For the formulation containing 0.5% PCL_5000_-PEG_5000_, as the PTX concentration increased up to 1.0 mg/mL, the surface density increased (from 2.1 to 3.0) and the packing parameter decreased (from 0.9 to 0.7) (Figure 4C). At PTX concentrations higher than 1.0 mg/mL, an opposite profile was observed. Thus, these changes suggest that increasing the PTX content to 1.0 mg/mL promotes the assembly of denser micellar shells, with PEG chains becoming more compact. Similar behavior was reported by Oda et al.,^31^ in which the presence of PTX in the core of polymeric micelles induced “shrinkage” of PEG chains. On the other hand, an inverted tendency was detected at formulations containing 1.0% PCL_5000_-PEG_5000_ (Figure 4D). In this series of samples, as the drug concentration increases a concomitant decrease in surface density of the shell chains from 2.5 to 1.0 and increase in packing parameter from 0.8 to 0.9 take place. Such condition is compatible with the center-to-center distance reduction, signaling smaller micelle cores and thicker (but less dense) micelle shells. The data suggest that increasing the PTX concentration destabilizes the formulation with the higher PCL_5000_-PEG_5000_ content, likely due to drug–copolymer competition within the micellar core.

Previous study has also showed that dense packing and strong entanglement of PEG chains can increase local hydrophobicity in the shell region, allowing hydrophobic molecules to penetrate and partially exclude water.^49^ In the present study, the higher PEG domain density observed in the 0.5% PCL_5000_-PEG_5000_ system suggests that increasing the PTX concentration may promote the accommodation of hydrophobic drug molecules at the core–shell interface, an effect that is less pronounced in the 1.0% formulation. To investigate this behavior, the impact of increasing drug concentrations on the aggregation number (*N*_agg_, the average number of molecules in a micelle), micelle core radius (*R*_c_), and micelle shell radius (*R*_s_) was evaluated from SAXS fits, with results summarized in Table 2.

**Table 2:** Structural Parameters of DSPE-PEG_2000_:PCL_5000_-PEG_5000_ Mixed Micelles, Including Aggregation Number (*N*_agg_), Core Radius (*R*_c_), and Shell Radius (*R*_s_), at Molar Ratios of 99.5:0.5 and 99:1 and Different PTX Concentrations

PTX concentration (mg/mL)	*N*_agg_	*R*_c_ (nm)	*R*_s_ (nm)
DSPE-PEG_2000_:PCL_5000_-PEG_5000_ (99.5:0.5)			
0	72.3	3.1	1.6
0.6	76.4	3.2	1.7
1.0	90.4	3.3	1.9
1.4	71.0	3.1	1.6
DSPE-PEG_2000_:PCL_5000_-PEG_5000_ (99:1)			
0	80.4	3.2	1.7
0.6	80.0	3.2	1.7
1.0	67.0	3.0	1.5
1.4	48.3	2.7	1.1

Differences between formulations with varying PCL_5000_-PEG_5000_ molar ratios were again evident. In the 0.5% PCL_5000_-PEG_5000_ formulation, values retrieved for *N*_agg_, *R*_c_, and *R*_s_ increased with PTX concentration up to 1.0 mg/mL, but all parameters decreased at 1.4 mg/mL, approaching values of micelles without PTX. In contrast, the 1.0% PCL_5000_-PEG_5000_ formulation showed a consistent decrease in these parameters starting at 1.0 mg/mL. Since *N*_agg_ is directly related to the micelle size by reducing interfacial free energy, these changes align with reports by Akiba et al.,^48^ which show that hydrophobic drug loading typically increases core size and the aggregation number. The parameter reductions observed at higher PTX concentrations in both formulations support the hypothesis of a micellar destabilization, indicating that the systems are unable to accommodate additional drug volume. A similar upper loading limit was reported by Oda and co-workers for DSPE-PEG_2000_ micelles, which began to destabilize above 0.6 mg/mL of PTX.^31^ Considering these findings and previous encapsulation efficiencies, 1.0 mg/mL PTX was selected for subsequent studies.

SAXS analysis thus provided structural insights into the mixed micellar systems, clarifying the influence of the secondary component (PCL_5000_-PEG_5000_) and the effects of increasing the drug concentration. The technique also supported the hypothesis that incorporating PCL_5000_-PEG_5000_, particularly at 0.5%, enhances drug loading capacity compared with DSPE-PEG_2000_ systems.^31^

### 4.4 Evaluation of Thermal Properties

Thermal analysis of the pure compounds and formulations was conducted using DSC, DTA, and TG/DTG for characterization and investigation of the possible interactions. For DSPE-PEG_2000_, the DSC curve (Figure 5A, in black) showed a characteristic endothermic melting peak at 46.8 °C (*T*_onset_), with an enthalpy change of 123.3 J g^–1^, as observed too in the DTA curve (Figure 5A, in green). This result is consistent with that previously reported by Tahir et al., who observed an endothermic melting peak near 50 °C for this component.^50^ In the TG curve (Figure 5A, in red), this material exhibited thermal stability up to 242.4 °C, above which a single-stage decomposition process begins, continuing to 437.1 °C and resulting in a total mass loss of 98.3% at the end of the analysis. DTG (Figure 5A, in pink) curves, for all analyses, supported the confirmation of the temperatures at which thermal events and mass variations occurred.

**Figure 5:**
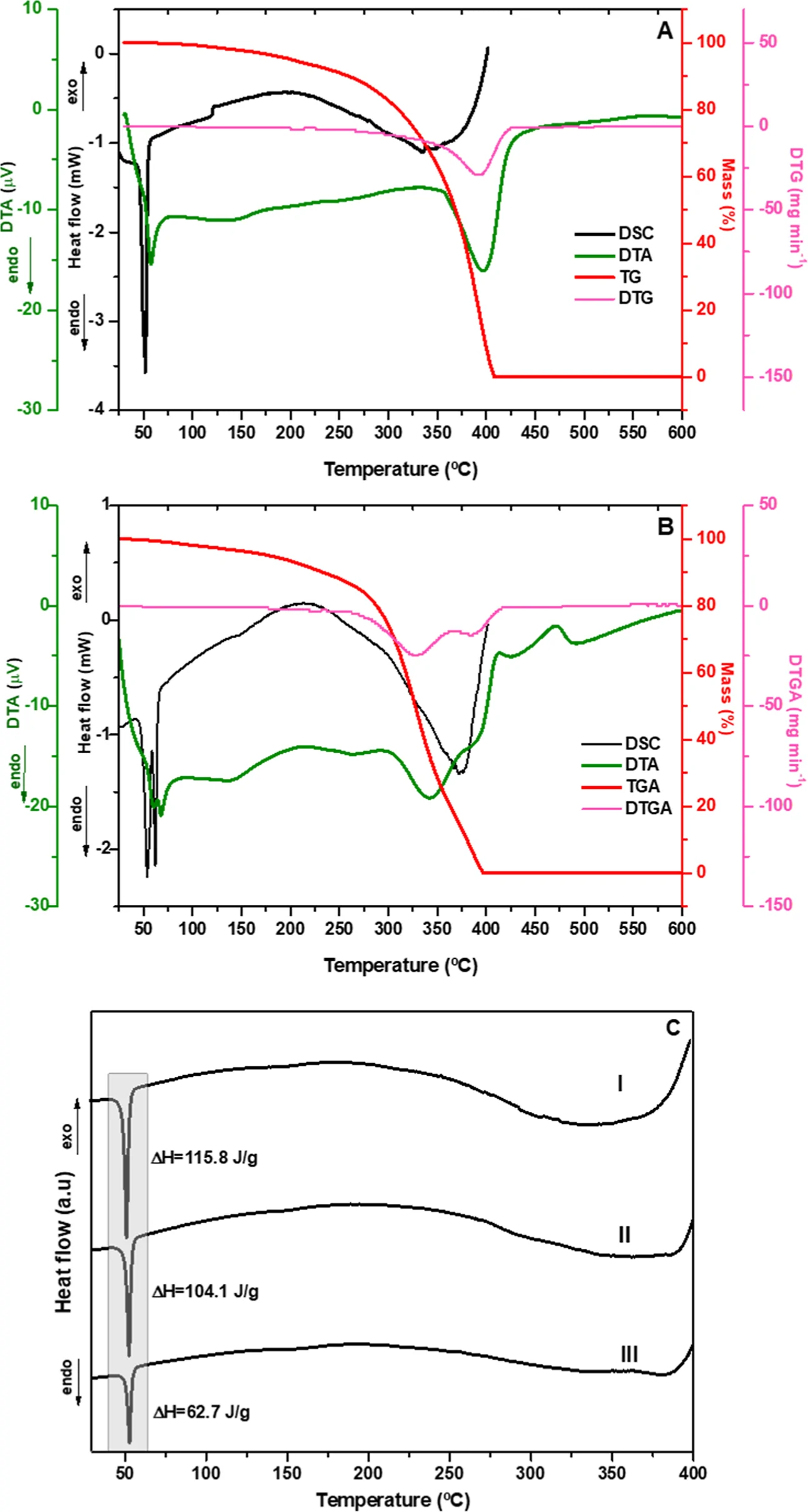
Thermal behavior of the raw material components DSPE-PEG_2000_ (A) and PCL_5000_-PEG_5000_ (B). (C) Thermal behavior evaluated by DSC of unloaded DSPE-PEG_2000_ micelle formulations (I) and unloaded mixed micelles of DSPE-PEG_2000_:PCL_5000_-PEG_5000_ at molar ratios of 99.5:0.5 (II) and 99:1 (III). The gray region highlights the endothermic melting peak.

For PCL_5000_:PEG_5000_, the DSC curve (Figure 5B, in black) displayed two closely spaced endothermic melting peaks characteristic of the copolymer, occurring at 49.4 °C (*T*_onset_) and 59.7 °C (*T*_onset_), confirming the contribution of the two constituent subunits (overlapping events in the DTA curve, Figure 5B, in green). The associated enthalpy changes were 60.7 J g^–1^ and 31.9 J g^–1^, respectively. Similar results were previously reported and endothermic melting peaks were observed near 60 °C for this component.^51,52^ Finally, TG data (Figure 5B, curve in red) showed thermal stability up to 240.9 °C for the copolymer, after which a two-stage decomposition process began: the first stage up to 357.3 °C, with a mass loss of 65.9%, and the second stage between 358.0 and 424.8 °C, with an additional mass loss of 27.8%, phenomena confirmed by the DTG curve (Figure 5B, in pink).

The thermal behaviors of the unloaded DSPE-PEG_2000_ micellar formulation (Figure 5C, curve I) and the micellar formulations composed of DSPE-PEG_2000_:PCL_5000_-PEG_5000_ at proportions of 0.5% and 1.0% were also evaluated (Figure 5C, curves II and III, respectively). The DSPE-PEG_2000_ micellar formulation showed a thermal behavior similar to that of the raw material, exhibiting a characteristic endothermic melting peak at 50.6 °C (*T*_onset_), with an enthalpy change of 115.8 J g^–1^. This result indicates that the micellization process, involving an organic solvent and heating, did not alter the thermal properties of the material, suggesting a reorganization event during micelle formation. In curve B, corresponding to the formulation containing 0.5% PCL_5000_-PEG_5000_, the endothermic melting peak occurred at 50.5 °C (*T*_onset_), with an enthalpy change of 104.1 J g^–1^. In curve C, for the formulation containing 1.0% PCL_5000_-PEG_5000_, the endothermic melting peak was observed at 50.8 °C (*T*_onset_), with an enthalpy change of 62.7 J g^–1^.

The progressive decrease in the required enthalpy is consistent with the increased proportion of PCL_5000_-PEG_5000_ in the system, a component that, in its raw material state, exhibited lower enthalpy changes during melting transitions (60.7 J g^–1^ and 31.9 J g^–1^) compared with DSPE-PEG_2000_ (123.3 J g^–1^). Thus, the presence of the copolymer exerts a facilitating effect on the phase-transition mechanism in mixed micellar systems relative to the conventional micellar system.

Concerning the TG curve of the DSPE-PEG_2000_ formulation (Figure 6, in black), an initial dehydration process was observed, with a mass loss of approximately 9.3% up to 216.9 °C. At this temperature, a single-stage decomposition process began and continued until 426.1 °C, resulting in an additional mass loss of 81.2%. For the component in its raw material state, the decomposition process started at a higher temperature (242.4 °C) and occurred in a single stage. For mixed micelle formulation containing 0.5% PCL_5000_-PEG_5000_ (red curve), a two-stage decomposition process was observed. The initial dehydration led to a 6.8% mass loss up to 154.8 °C. Then, the first decomposition stage occurred, ending at 262.1 °C and causing a 10.1% mass loss. The second stage, from 264.6 to 442.1 °C, resulted in an additional 72.4% mass loss. Increasing PCL_5000_-PEG_5000_ to 1.0% (blue curve) also led to a two-stage decomposition process. The initial dehydration resulted in a 5.6% mass loss. The first decomposition stage occurred between 168.0 and 286.1 °C, resulting in an additional 25.1% mass loss. The second stage, from 287.2 to 474.2 °C, resulted in an additional 52.5% mass loss. The thermogravimetric analysis allowed assessment of the thermal stability and decomposition behavior of the formulations, revealing distinct patterns. A leftward shift in the curves was observed, indicating that decomposition events began at lower temperatures than with the conventional formulation. Furthermore, the presence of PCL_5000_-PEG_5000_ led to two-stage decomposition in the mixed micelle systems, reflecting the structural contribution of this copolymer.

**Figure 6:**
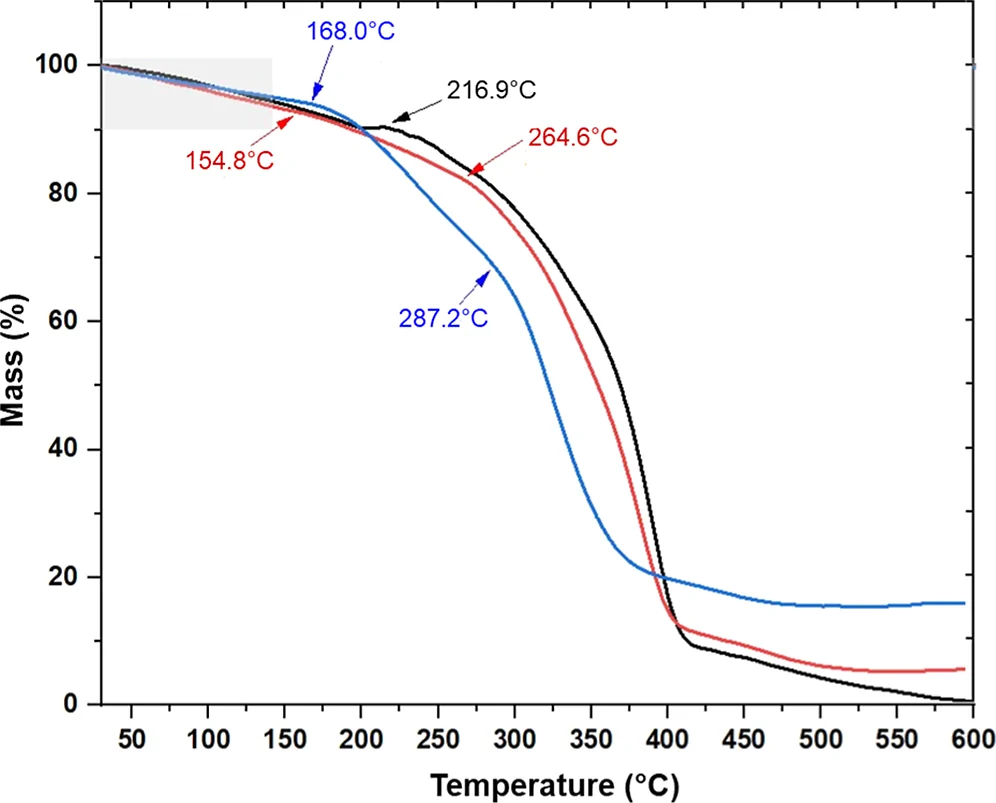
Thermal behavior evaluated by TGA of unloaded PTX micelle formulations of DSPE-PEG_2000_ (black curve), DSPE-PEG_2000_:PCL_5000_-PEG_5000_ at a molar ratio of 99.5:0.5 (red curve), and DSPE-PEG_2000_:PCL_5000_-PEG_5000_ at a molar ratio of 99:1 (blue curve). The gray box indicates the expected dehydration region of the samples, corresponding to moisture loss and volatilization of components. Arrows in the respective colors indicate the start of the thermal decomposition events of the samples.

### 4.5 Stability Study

Polymeric micelles behave as dynamic structures whose integrity can be affected by small changes in the environments in which they are placed.^53,54^ To evaluate the behavior of the mixed micelles produced, a stability study was conducted by monitoring diameter, zeta potential, and encapsulation efficiency. The results are presented in Figure 7. The mean diameters obtained for the formulations containing 0.5% or 1.0% of the copolymer throughout the study period were 11.5 ± 0.5 nm and 11.6 ± 0.8 nm, respectively. At each fixed time point, no significant differences were observed between the formulations with higher or lower copolymer content. The mean zeta potential values obtained over the course of the study for the formulations containing 0.5% or 1.0% of the copolymer were −3.7 ± 1.0 mV and −3.1 ± 0.4 mV, respectively. For the formulation at the 99.5:0.5 molar ratio, this parameter showed a significant change compared with the day of preparation and with the formulation at the 99:1 molar ratio only on the seventh day of analysis. For the remaining time points, no significant differences were observed. For the formulation at the 99:1 molar ratio, no significant differences in this parameter were found at any time point compared with the day of preparation. Overall, the differences in diameter and zeta potential were not considered sufficient to indicate instability of the system, as the diameter remained lower and the zeta potential remained close to neutrality.

**Figure 7:**
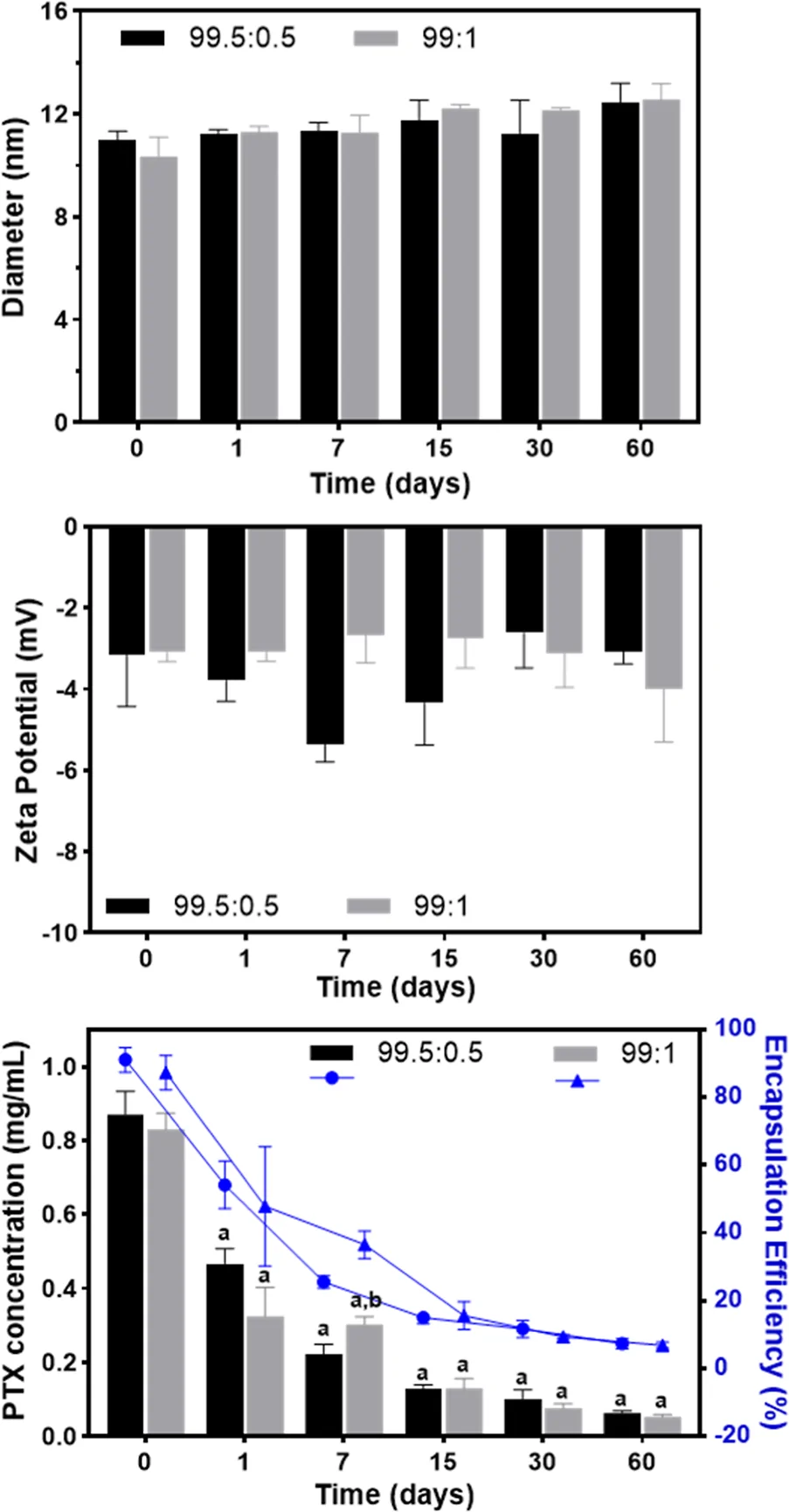
Time-dependent stability study of mixed micelles composed of DSPE-PEG_2000_:PCL_5000_-PEG_5000_ at molar ratios of 99.5:0.5 and 99:1, evaluating mean hydrodynamic diameter (number-weighted analysis), zeta potential, encapsulated PTX concentration, and encapsulation efficiency.

Regarding encapsulation efficiency, although high encapsulation levels were achieved on the day of preparation for both formulations, it was not possible to observe a drug-retention profile in the mixed micelle system over the evaluated period. All time points showed significant differences compared with the day of preparation. A significant difference (*p* ≤ 0.05) was observed only on the seventh day of analysis, in which the mixed micelle formulation at the 99:1 molar ratio retained an average of 36.5 ± 4.1% of the encapsulated drug, whereas the 99.5:0.5 formulation retained an average of 25.5 ± 1.8%. Oda (2015) reported an average drug retention of 14.0 ± 5.3% on the seventh day of analysis for DSPE-PEG_2000_ micelles at the same theoretical initial PTX concentration of 1.0 mg/mL. These results suggest that incorporating the PCL_5000_-PEG_5000_ copolymer into the mixed micelle formulations did not provide a meaningful improvement in micellar stability over time under the conditions evaluated in this study. However, an increase in drug retention up to the seventh day was observed compared with the previously developed system lacking the copolymer.

The low stability of polymeric micellar systems in predominantly aqueous environments is a well-known phenomenon and one of the most relevant limitations to the efficiency of these systems, with polymer concentration, molecular weight of the core-forming block, drug incorporation, and drug–core compatibility being examples of factors capable of influencing this phenomenon.^50–56^ Block copolymers such as PCL-PEG and DSPE-PEG contain an ester group susceptible to hydrolysis, located between the hydrophilic and hydrophobic segments. In core–shell micelles, this group is positioned at the interface with the medium, making it readily accessible to reaction. Hydrolysis of this interfacial ester results in detachment of the PEG block and destabilization of the micellar system.^56^ Additionally, monomer desorption is an activated micelle-destabilization process in which a high-energy transition state is formed when most of the hydrophobic portion of the monomer becomes exposed to the surrounding solvent, while the monomer remains anchored to the micelle by only a small segment of its chain. After detaching from the micelle core, the monomer gains rotational and translational degrees of freedom, thereby reducing its overall energy. Kinetic data suggest that this phenomenon occurs even at low temperatures, such as 5 °C.^25^ To verify the occurrence of this and other mechanisms, additional analyses would be required, which were not performed in the present study.

Additionally, to assess the colloidal stability of the formulations under biologically relevant conditions, micelles’ diameter was monitored in FBS and RPMI 1640 complete culture medium over 24 h (Figure 8). The evaluation of stability under physiological conditions is essential for micellar drug delivery systems, as these nanocarriers must preserve their structural integrity and retain the encapsulated drug during circulation.^57^ Although minor fluctuations in hydrodynamic diameter were observed over time, both maintained diameters within the nanoscale range (approximately 5–15 nm), demonstrating satisfactory colloidal stability in the tested biological media.

**Figure 8:**
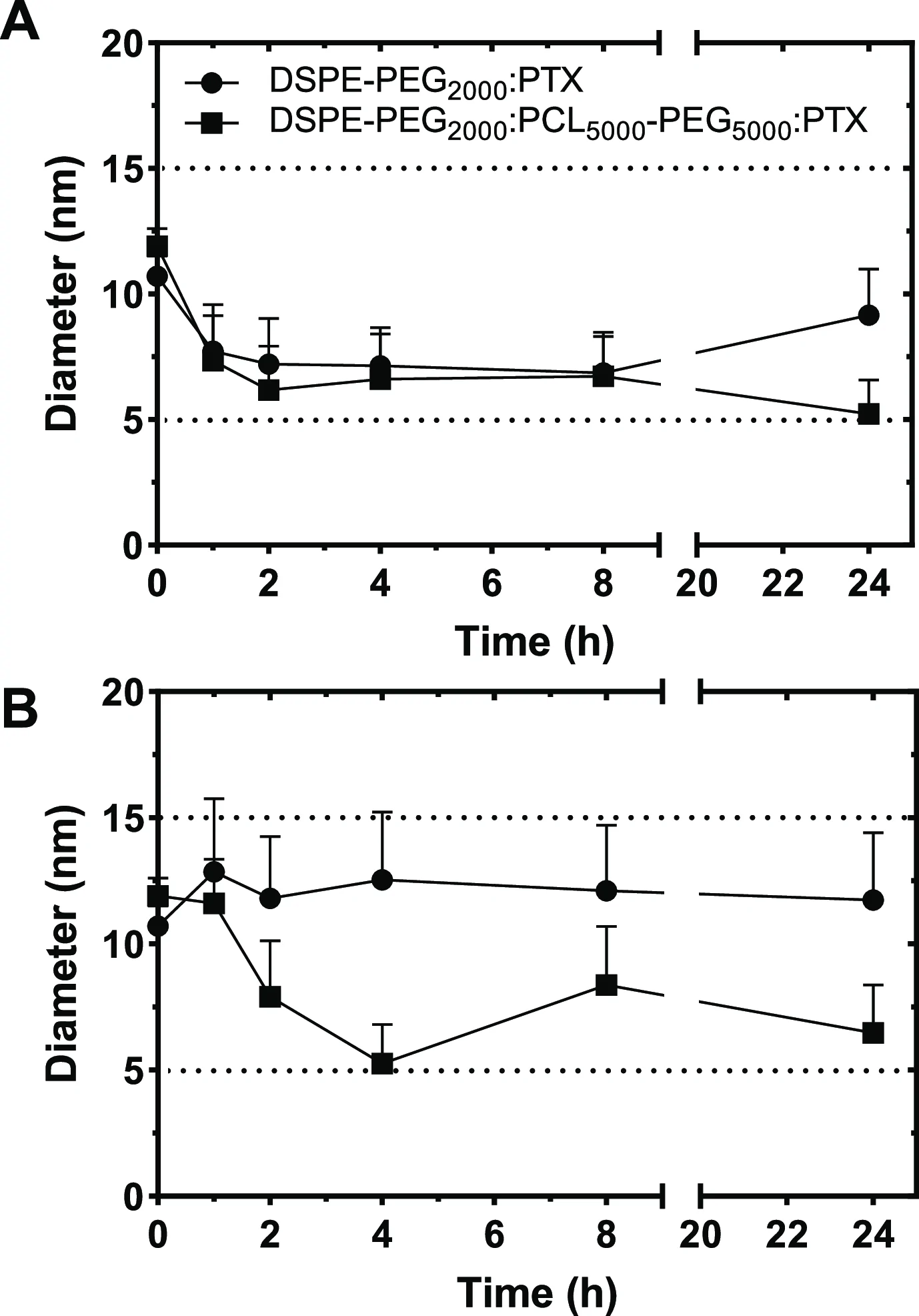
Temporal evolution of average diameter in fetal bovine serum (A) and RPMI 1640 (B).

### 4.6 Release Study

The in vitro drug release profile is crucial for developing effective drug delivery systems. However, for highly hydrophobic drugs such as PTX, achieving and maintaining sink conditions, indispensable for the release study, is a major challenge. To address this issue, several solubility modifiers, including surfactants, inorganic salts, proteins, and organic solvents, have been reported in in vitro release studies of polymeric PTX carriers.^58^

In this study, we used sodium salicylate as a hydrotropic agent in the release medium, as previously described.^22^ Hydrotropic agents are ionic organic salts that increase the solubility of hydrophobic compounds through weak interactions.^59,60^ Compared to surfactants, hydrotropes have a smaller hydrophobic part, making them more suitable for certain applications.^60^

In the present work, PTX solubility in 1 mol/L sodium salicylate was 32.9 ± 4.8 μg/mL after 24 h of incubation, consistent with the previously reported value of 28.1 μg/mL.^22^ We also evaluated the hydrodynamic diameter of the micelles, which remained unchanged in the presence of the hydrotropic agent. These results confirmed the suitability of sodium salicylate as a release medium. As no relevant difference in physicochemical properties or stability was observed between mixed micelles containing 0.5% or 1.0% PCL_5000_=PEG_5000_, the formulation with the lower copolymer content was selected for the release study and compared with DSPE-PEG_2000_ micelles. The percentage of PTX released as a function of time is shown in Figure 9. No significant difference was observed for the release profiles of the DSPE-PEG_2000_ and DSPE-PEG_2000_:PCL_5000_-PEG_5000_ (99.5:0.5) formulations, indicating that, at a molar ratio of 0.5%, the addition of the PCL_5000_-PEG_5000_ copolymer did not significantly affect drug release from the mixed micelle system.

**Figure 9:**
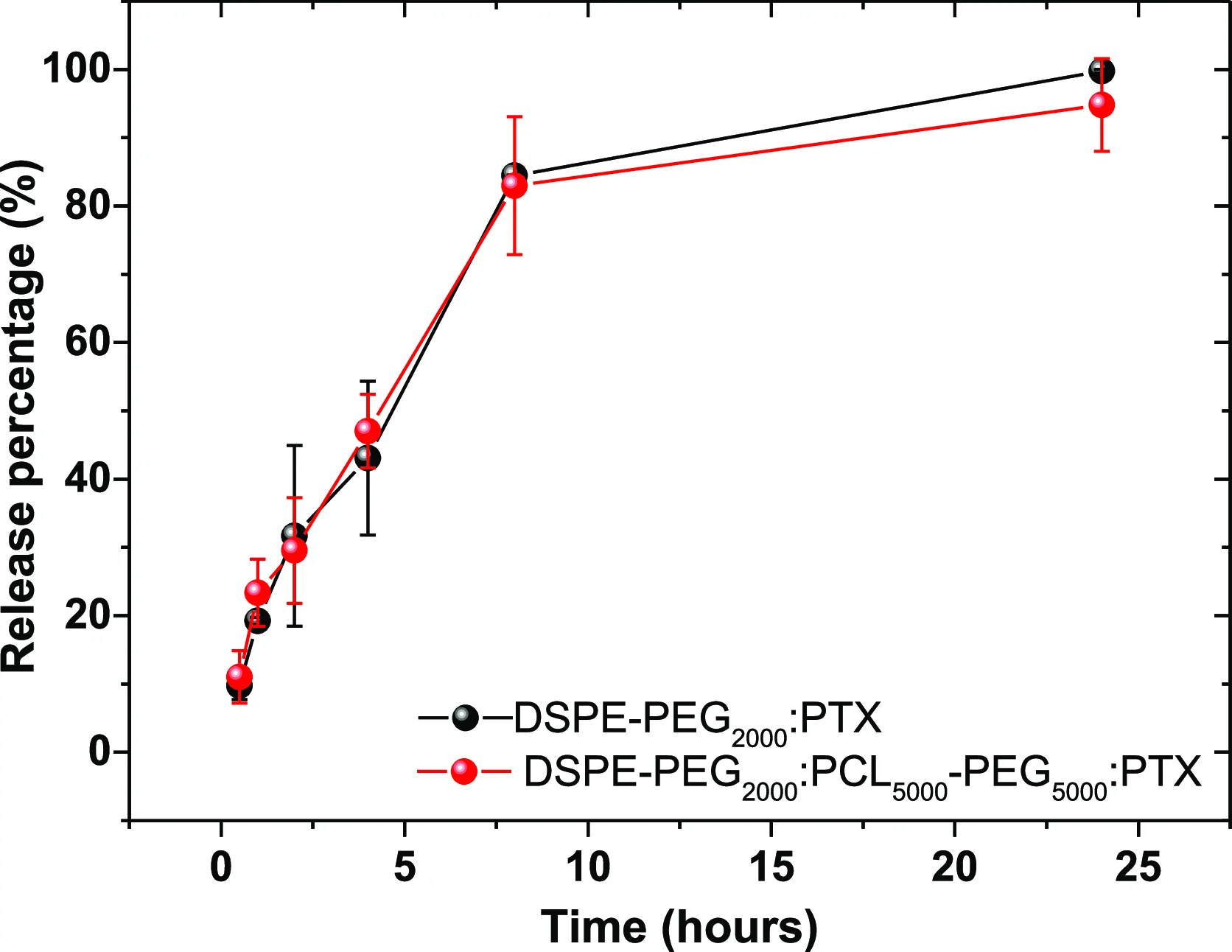
Cumulative percentage of PTX released versus time for DSPE-PEG2000 formulations (0.6 mg/mL of PTX) and DSPE-PEG_2000_:PCL_5000_-PEG_5000_ at a molar ratio of 99.5:0.5 (1.0 mg/mL of PTX) in 1 mol/L sodium salicylate solution at 37 °C. Data are presented as mean ± the standard deviation of the mean (*n* = 3).

The biphasic release profile observed for the mixed micelles is consistent with the behavior commonly reported for polymeric micellar systems, in which an initial burst release is followed by a sustained diffusion-controlled release from the hydrophobic core.^14^ The initial release phase is attributed to the rapid diffusion of PTX molecules weakly associated with the micellar surface, which are more readily accessible to the release medium. In contrast, the second release stage is governed by the diffusion of PTX molecules entrapped within the micelles’ hydrophobic core. Strong hydrophobic interactions between PTX and the polymers’ hydrophobic domains contribute to drug retention within the micellar structure, resulting in a slower, more sustained release rate.

### 4.7 Cytotoxicity Study

The cytotoxic activity of PTX-loaded micelles was evaluated in 4T1 murine breast cancer cells. The 4T1 breast cancer model is widely used as a representative model of human triple-negative breast cancer because it reproduces several key features of this disease, including aggressive tumor progression, local invasion, and spontaneous metastasis to distant tissues.^61^ The cytotoxicity results are presented in Figure 10. Both micellar formulations exhibited potent antiproliferative effects with IC50 values of 81.8 nM for DSPE-PEG_2000_ micelles and 79.7 nM for DSPE-PEG_2000_:PCL_5000_-PEG_5000_:PTX micelles. These values were comparable to those obtained for Taxol (109.6 nM) since the comparison among fitting curves showed a P value equal to 0.73. These data indicate that PTX retained its biological activity following encapsulation within micellar systems. The slightly lower IC_50_ values observed for the micellar formulations suggest that the nanoformulations effectively delivered PTX to tumor cells while preserving their antitumor efficacy.

**Figure 10:**
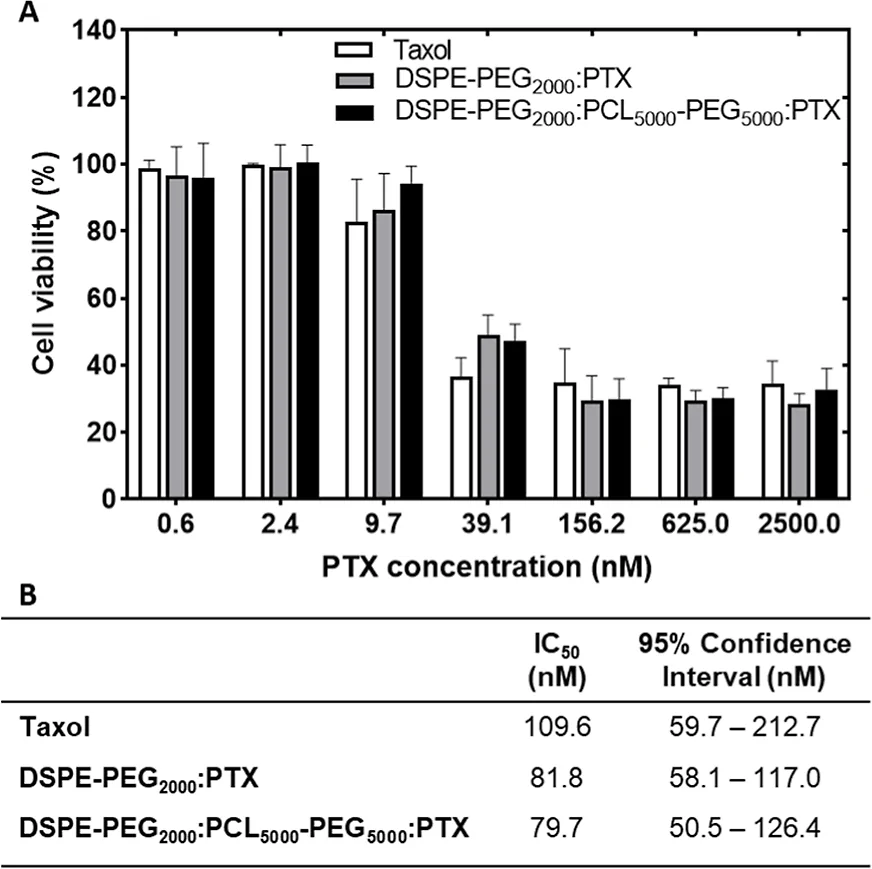
In vitro cytotoxicity of PTX-loaded micellar formulations in 4T1 murine breast cancer cells. (A) Cell viability determined by the sulforhodamine B (SRB) assay after 48 h of treatment with DSPE-PEG_2000_:PTX micelles and DSPE-PEG_2000_:PCL_5000_-PEG_5000_:PTX micelles at different paclitaxel concentrations. (B) IC_50_ values and corresponding 95% confidence intervals obtained from the dose–response curves. Data are expressed as mean ± SD of three independent experiments performed in triplicate.

### 4.8 Hemolytic Study

The hemolytic activity of DSPE-PEG_2000_:PCL-PEG_5000_ was evaluated in vitro using blood compatibility testing, given the known hemolytic effects associated with both PTX and the Cremophor-based commercial formulation. The DSPE-PEG_2000_:PCL_5000_-PEG_5000_ 99.5:0.5 formulation was selected for this study, and two PTX concentrations (0.05 and 0.10 mg/mL) were investigated. The hemolytic profile of the mixed micelles was compared with that of a Cremophor:ethanol:PTX formulation prepared similarly to the commercial product (Taxol) and DSPE-PEG_2000_ micelles.

At PTX concentrations of 0.05 and 0.10 mg/mL, the Cremophor:ethanol:PTX formulation induced significant hemolysis, reaching approximately 6% and 16%, respectively, consistent with known membrane-disruptive effects. In contrast, PTX-loaded DSPE-PEG_2000_ and DSPE-PEG_2000_:PCL_5000_-PEG_5000_ micelle formulations showed significantly lower hemolytic activity compared to the Cremophor:ethanol:PTX formulation (Figure 11). According to the ASTM E2524-08 standard,^62^ nanoparticulate systems exhibiting hemolysis below 2% are classified as nonhemolytic. Thus, the mixed micelles developed in this study can be considered nonhemolytic and biocompatible. These findings suggest that the incorporation of PTX into mixed micelles effectively reduces its hemolytic toxicity, supporting the potential of this delivery system as a safer alternative to Cremophor-based formulations for intravenous cancer chemotherapy.

**Figure 11:**
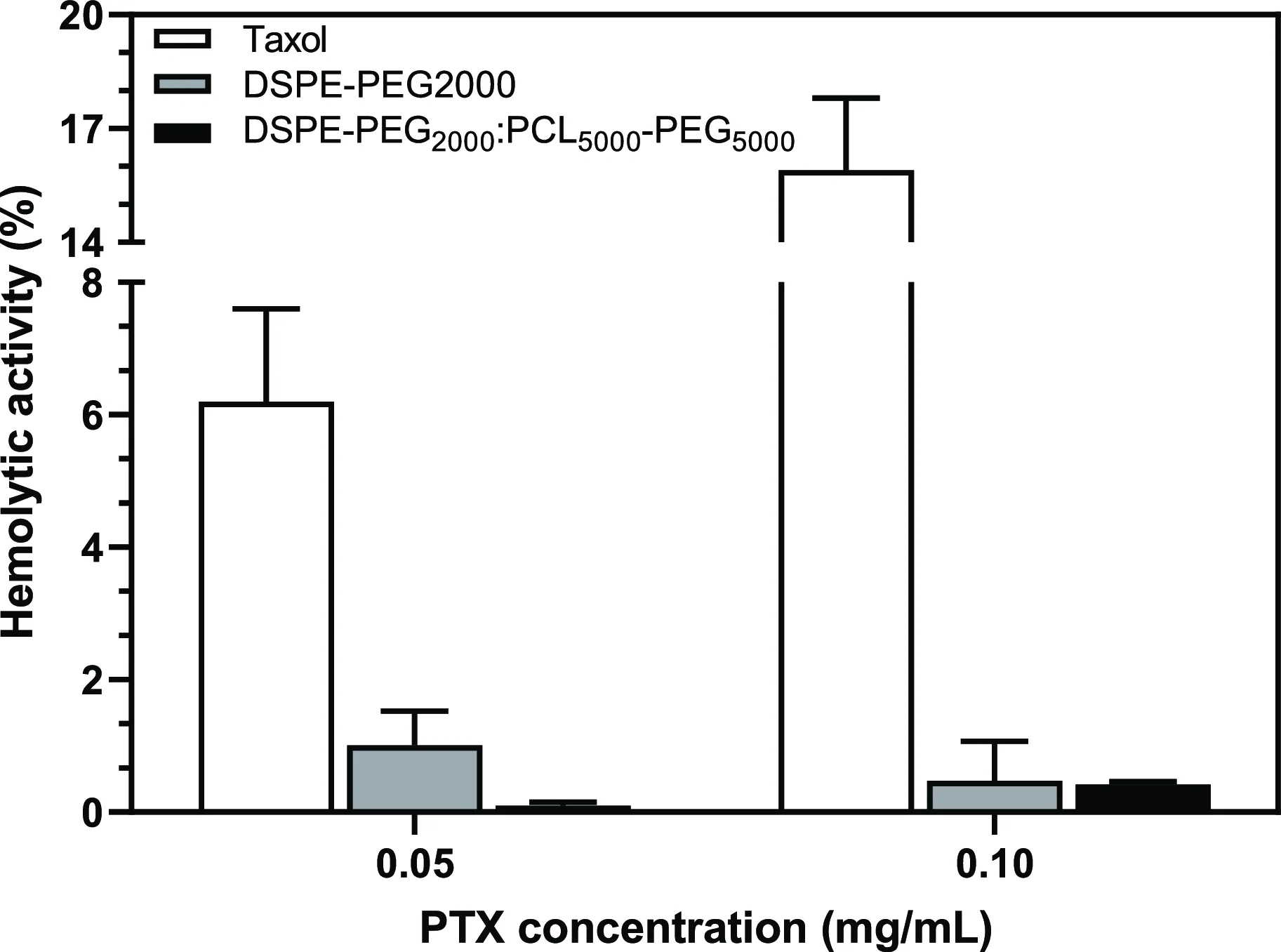
Evaluation of the percentage of hemolysis of red blood cells induced by PTX-loaded DSPE-PEG_2000_ or DSPE-PEG_2000_:PCL_5000_-PEG_5000_ (at a molar ratio of 99.5:0.5) micelles compared to Cremophor:ethanol:PTX (similar formulation to Taxol). Data are presented as mean ± standard deviation of the mean (*n* = 5).

## 5 Conclusions

In summary, this study developed mixed micelles composed of DSPE-PEG_2000_ and PCL_5000_-PEG_5000_ with a diameter of approximately 11 nm and a drug loading capacity of 3.1% w/w. The formulation was nonhemolytic, providing a biocompatible alternative to the toxic surfactants used in some commercial products. Although aqueous stability remains a challenge, the improved drug retention and favorable core–shell architecture highlight the potential of these mixed micelles as a safer platform for intravenous cancer chemotherapy.

Despite these promising findings, including nanoscale size, high encapsulation efficiency, cytotoxic activity comparable to that of commercial formulations, and reduced hemolytic toxicity, several limitations must be addressed before clinical translation can occur. The present work was limited to formulation development and physicochemical characterization; therefore, the in vivo behavior remains to be determined. In addition, challenges associated with large-scale manufacturing and regulatory approval must be considered. Accordingly, further preclinical investigations are required to validate the translational potential of these mixed micelles and assess their suitability for future clinical applications.
